# Gene expression patterns associated with caudal fin shape in the cichlid *Lamprologus tigripictilis*

**DOI:** 10.1007/s10750-022-05068-4

**Published:** 2022-11-18

**Authors:** Ehsan Pashay Ahi, Florian Richter, Kristina M. Sefc

**Affiliations:** 1 Institute of Biology, University of Graz, Universitätsplatz 2, 8010 Graz, Austria; 2 Organismal and Evolutionary Biology Research Programme, University of Helsinki, Viikinkaari 9, 00014 Helsinki, Finland

**Keywords:** Cichlidae, Lamprologini, Fin morphogenesis, Fin regeneration, Fin growth, Morphological diversity

## Abstract

Variation in fin shape is one of the most prominent features of morphological diversity among fish. Regulation of fin growth has mainly been studied in zebrafish, and it is not clear whether the molecular mechanisms underlying shape variation are equally diverse or rather conserved across species. In the present study, expression levels of 37 candidate genes were tested for association with fin shape in the cichlid fish *Lamprologus tigripictilis*. The tested genes included members of a fin shape-associated gene regulatory network identified in a previous study and novel candidates selected within this study. Using both intact and regenerating fin tissue, we tested for expression differences between the elongated and the short regions of the spade-shaped caudal fin and identified 20 genes and transcription factors (including *angptl5*, *cd63*, *csrp1a*, *cx43*, *esco2*, *gbf1*, and *rbpj*), whose expression patterns were consistent with a role in fin growth. Collated with available gene expression data of two other cichlid species, our study not only highlights several genes that were correlated with fin growth in all three species (e.g., *angptl5*, *cd63*, *cx43*, and *mmp9*), but also reveals species-specific gene expression and correlation patterns, which indicate considerable divergence in the regulatory mechanisms of fin growth across cichlids.

**Supplementary Information:**

The online version contains supplementary material available at 10.1007/s10750-022-05068-4.

## Introduction

The fins of fish contribute to various functions such as locomotion and communication (Wainwright et al., 2002; Lönnstedt et al., 2014), and variation in fin shape is one of the most prominent features of morphological diversity among fish species (e.g., Gilbert et al., 2022). In ray-finned fishes, the shape of the fins is determined predominantly by variation in the length of the individual fin rays. In many species, one or more of the fins are adorned by elongated filaments, which are formed by accelerated or prolonged growth of these fin regions during larval or later development. Less conspicuous shapes, such as spade- of fork-shaped caudal fins, are likewise the product of ray length variation within fins. Importantly, fin shape can regenerate completely after damage or experimental amputation throughout the lifetime of the fish, suggesting that it is under rather strict genetic control and that positional memory orchestrates the molecular factors necessary for fin regeneration (Rabinowitz et al., 2017). The fin rays of teleost fish are segmented, and both ontogenetic and regenerative growth involves the addition of bony segments at their distal ends. Ray length is therefore determined by both the length and the number of individual segments. At the molecular level, this involves components of various developmental pathways, such as WNT, FGF, Hedgehog, and retinoic acid (RA) signaling, as well as epigenetic, skeletogenic, and structural remodeling factors (Iovine, 2007; Yoshinari et al., 2009; Wehner & Weidinger, 2015; Sehring & Weidinger, 2020; Singh et al., 2021). Most of the ground-laying work on the genetic control of fin growth and shape has concentrated on zebrafish (Pfefferli & Jaźwińska, 2015), and it is not clear to which degree the molecular and anatomical basis of fin shape formation varies among fish species.

We have previously conducted a study of the anatomy and the molecular mechanisms underlying the ornamental fin shape of the cichlid fish *Neolamprologus brichardi* (Trewavas & Poll, 1952), an endemic of Lake Tanganyika in East Africa (Ahi et al., 2017; Ahi & Sefc, 2018). In this species, the distal tips of the dorsal and anal fins as well as the dorsal and ventral tips of the fork-shaped caudal fin are conspicuously elongated. Expression levels of candidate genes were compared between elongated and non-elongated (short) regions within the same fins, using intact as well as regenerating fin tissue sampled from adult fish. The observed gene expression patterns and correlations led to the proposition of a gene regulatory network (GRN) involved in the formation of the fin phenotype, hitherto referred to as *N.b.*-GRN. Members of the *N.b.*-GRN include genes reported to be involved in fin ray segmentation, angiogenesis, or neurogenesis such as *cx43*, *mmp9*, *angptl5*, *angptl7*, *dpysl5a*, *csrp1a*, and *cd63* (Iovine et al., 2005; Monaghan et al., 2006; Nakatani et al., 2007; Sims et al., 2009; Ma et al., 2012; Ton & Iovine, 2012, 2013; Kang et al., 2015; Hagedorn et al., 2016), and several potential upstream regulators for this gene network including *egr2*, *foxc1*, *foxd3*, *foxp1*, *irf8*, and *myc* (Ahi & Sefc, 2018). Among the latter, *myc*, *irf8*, and *foxd3* were already indicated in fin regeneration studies of other teleost fish (Christen et al., 2010; Li et al., 2012; Kang et al., 2015; Hasegawa et al., 2017; Huang & Chen, 2017). We predicted *foxd3* as the key upstream regulator of the gene network in *N. brichardi*, since it consistently displayed significant expression correlation with all members of the network genes (Ahi & Sefc, 2018).

We next investigated whether members of the *N.b.*-GRN were also implicated in similar elongations of the dorsal and anal fins in another East African cichlid species, *Steatocranus casuarius* (Poll, 1939)*,* a member of Steatocranini tribe which is a sister lineage to the Lake Tanganyika cichlids (Ahi et al., 2019a). Only a subset of the tested genes showed the expected expression level differences between short and elongated fin regions, indicating that the molecular mechanisms controlling fin elongation differed between the two cichlid species. The divergence in the molecular mechanisms of fin elongations between the two species was accompanied by anatomical differences. In *N. brichardi*, fin ray segments in the elongated fin regions were shorter or the same length as the fin ray segments in the short fin regions, suggesting that the elongation must be due to a larger number of segments in the elongated rays (Ahi et al., 2017). In *S. casuarius*, in contrast, segments of the elongated rays were longer than the fin ray segments in the short fin region (Ahi et al., 2019a).

With a divergence time of about 14 million years between their respective tribes (Irisarri et al., 2018), *N. brichardi* (tribe Lamprologini) and *S. casuarius* (tribe Steatocranini) represent two rather distantly related cichlids with convergent shapes of the dorsal and anal fins (Ahi et al., 2019a). In the present study, we ask whether the genetic control of fin growth is more conserved between two less divergent cichlid species. To this aim, we examine the caudal fin of *Lamprologus tigripictilis* (Schelly & Stiassny, 2004), which is reverse to the fork-shape of the caudal fin in *N. brichardi*—specifically, a spade-shaped caudal fin, in which the medial region is elongated compared to the short dorsal and ventral regions of the fin. *L. tigripictilis* is also a member of the Lamprologini and considerably more closely related to *N. brichardi* than *S. casuarius*, as the radiation of the Lamprologini started at only about 6 million years ago (Irisarri et al., 2018). If mechanisms of fin growth are shared between the two lamprologine cichlids, we expect that many of the *N.b.*-GRN member genes will show corresponding gene expression differences between the elongated and short caudal fin regions in *L. tigripictilis*. We first examined the caudal fin expression patterns of 16 members of the *N.b.*-GRN by comparing gene expression levels in short and elongated regions using qPCR. Since only few of the *N.b.*-GRN showed the expected expression patterns, we then expanded the set of candidate genes based on co-expression data and transcription factor prediction and tested their expression in the fin tissue samples of *L. tigripictilis*. We also measured the length of the fin ray segments in the short and elongated fin rays in order to assess the anatomical basis of fin elongation in *L. tigripictilis*.

## Methods

### Fin sampling for RNA isolation

**Fig. 1:**
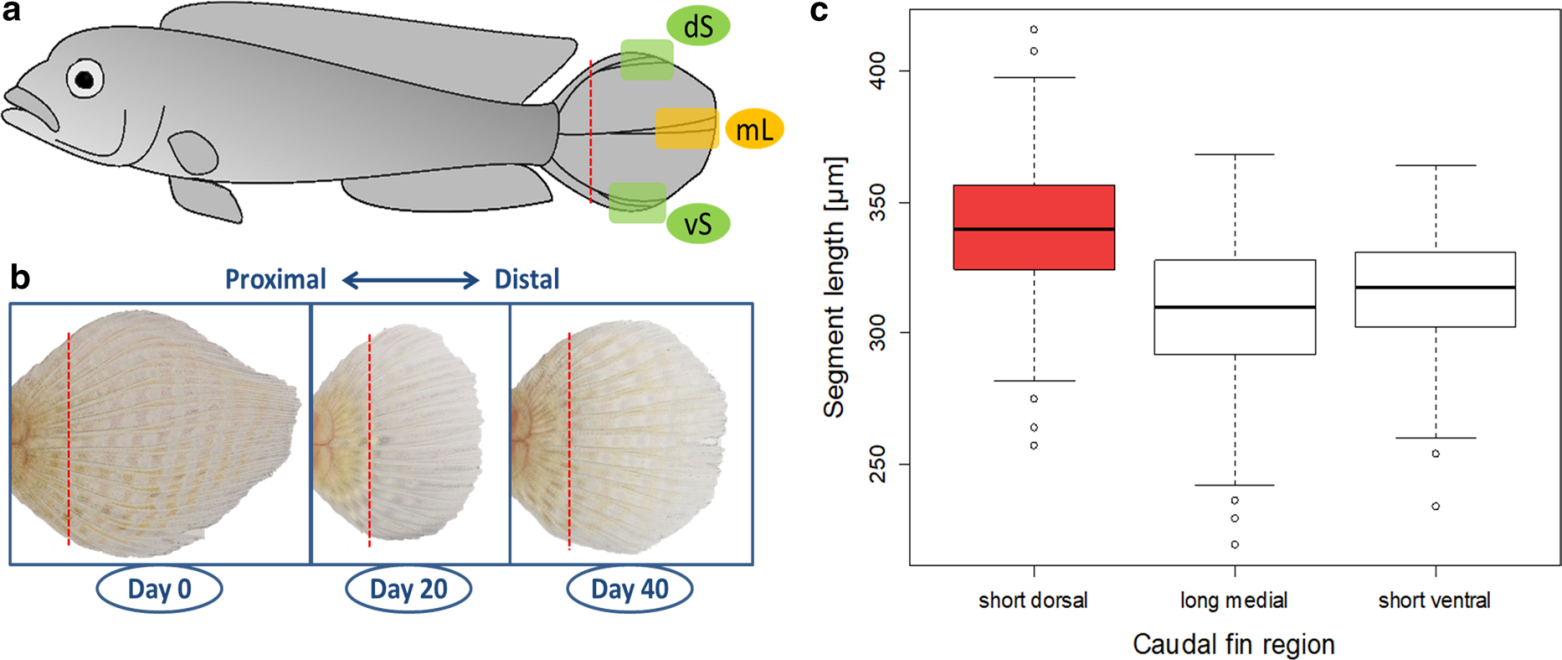
The caudal fin of *L. tigripictilis*. **A** Caudal fins were amputated along the dashed red line, and tissue samples representing the short dorsal (dS), the short ventral (vS), and the elongated medial (mL) regions of the dorsal fin were taken as indicated. **B** Biopsies were taken from the original fin tissue (day 0, representing stage 0) and from regenerating tissue at day 20 (representing stage 1) and day 40 (representing stage 2) after the preceding amputation. **C** Length of fin ray segments in the three regions of the caudal fin. Different box colors indicate significant differences in segment length

We used six captive bred adult males of *L. tigripictilis* (total length 7–10 cm). Prior to taking the fin biopsies, fish were anesthetized using 0.04 g of MS-222 per liter of water. Then, the caudal fin was cut in front of the first ray bifurcation (branching) under a stereomicroscope (red dashed lines in Fig. 1A, B). Next, three separate tissue samples were dissected from the severed fin (Fig. 1A): one comprised the most dorsal, branched fin ray and represented the dorsal short fin region (dS), one comprised the most ventral, branched fin ray and represented the ventral short fin region (vS), and one comprised the medial fin ray and represented the medial elongated fin region (mL). Each sample comprised the two branches of the selected fin ray. The tissue samples were stored frozen in RNA later (Qiagen) until RNA isolation. Biopsies were taken from the original fin tissue (stage 0) to study gene activity patterns associated with the maintenance of the phenotype, and then twice during regeneration, including a biopsy at day 20 after the first biopsy when the round shape of the fin started to appear (stage 1), and another biopsy at day 40 after the stage 1 biopsy, when the fin had become distinctly rounded (stage 2). The biopsy regime is illustrated in Fig. 1B. Anesthesia and fin biopsies were performed under permit number BMWFW-66.007/0028-WF/V/3b/2017 issued by the Federal Ministry of Science, Research and Economy of Austria (BMWFW). All methods were performed in accordance with the relevant guidelines and regulations of BMWFW.

### Fin ray segment length measurements

To measure the length of fin ray segments in elongated medial and the short dorsal and ventral regions of the caudal fin, biopsies were taken from six adult males in the same way as fin sampling for RNA isolation at stage 0 described above and stained with alizarin red. We modified the acid-free double staining protocol described by Walker & Kimmel (2007) and used 10% KOH in the clearing phase, increased the duration of the staining phase to 4 days and the duration of the clearing phase to 15 days. Using a Keyence VHX-5000 Digital Microscope, we measured the length of the 10 most distal, complete segments on one branch per each of two dorsal (short), two ventral (short), and four medial (elongated) fin rays. That is, measurements were taken from eight different rays, and not from branches pertaining to the same ray. The selected fin rays were the two most dorsal, branched rays, the two most ventral, branched rays (we did not use the most dorsal and the most ventral fin ray, as these were only rudimentary developed in some individuals) and the four medial fin rays. The branch representing each fin ray was either selected randomly or by avoiding irregularities in the segmentation pattern, which sometimes occurred in one of the branches. We used a linear mixed model (R package lmerTest) to compare segment length between the three fin regions, including segment length as response variable and fin region as predictor variable. To account for possible correlations within rays and individual fins, we included ‘fin ray’ nested in biological replicate (fish) as random factors (Supplementary Data 2, Table S1).

### Candidate target and reference gene selection

The selection and analysis of candidate target genes and transcription factors (TF) was performed in three steps (see Ahi et al., 2015). First, we tested 16 genes of the *N.b.*-GRN described in the introduction (Ahi & Sefc, 2018). These genes were *angptl5*, *angptl7*, *anxa2a*, *c1qtnl5a*, *cd63*, *csrp1a*, *cx43*, *dpysl5a*, *gnao1a*, *kif5a*, *mmp9*, *pfkpa*, *sema3d*, *txn*, *wnt10a*, and *wnt5b*. Out of the first set of candidate genes, we picked the ones with the strongest expression differences between the elongated and the short fin regions (*angptl5*, *cd63*, *csrp1a*; see “Results” section) to search for genes co-expressed with all the three genes (top overlapping co-expressed genes) in the zebrafish co-expression database, COXPRESdb (http://coxpresdb.jp/) version 6.0 (Obayashi & Kinoshita, 2011). To attain a high degree of reliability, we filtered the genes co-expressed with each of the three genes by setting the Supportability score to a minimum of 1 (as described by Obayashi & Kinoshita, 2011) (Supplementary data 1). This step identified eight additional candidate genes (see results). Finally, we selected eight of the above 24 candidate genes, namely those with strongly increased expression in the elongated fin region (see results), for TF prediction. In order to predict the potential upstream regulators for these genes, we performed motif enrichment on 4 kb upstream sequences (promoter and 5’-UTR) of these genes as previously described by (Lecaudey et al., 2019, 2021) using the annotated genome of the Nile tilapia, *Oreochromis niloticus* (Flicek et al., 2013), and two algorithms: MEME (Bailey et al., 2009) and XXmotif (Luehr et al., 2012). The motifs that were present in the promoters of at least half of these genes were compared to position weight matrices (PWMs) from the TRANSFAC database (Matys et al., 2003) using STAMP (Mahony & Benos, 2007) to identify matching transcription factor (TF) binding sites (Supplementary data 1).

To identify stable reference genes, we selected 8 candidate reference genes with abundant expression in a range of tissues, which have already been investigated as reference genes in fins or other tissues containing skeletal structures or/and epidermis in fish (Table 1). Candidate reference genes were ranked according to expression stability by three different algorithms, BestKeeper (Pfaffl et al., 2004), NormFinder (Andersen et al., 2004), and geNorm (Vandesompele et al., 2002). The standard deviation (SD) based on Cq values of the fin regions was calculated by BestKeeper to determine the expression variation for each reference gene. In addition to ranking, BestKeeper also determines the stability of reference genes through a correlation calculation or BestKeeper index (*r*). GeNorm calculates mean pairwise variation between each gene and other candidates (the expression stability or *M* value) in a stepwise manner and NormFinder identifies the most stable genes (lowest expression stability values) based on the analysis of inter- and intra-group variation in expression levels variations (Ahi et al., 2018, 2019b). All three algorithms ranked *rps18* and *actb1* as the two most stable candidate reference genes (Table 1). Based on these results, we used the geometric mean of the expression of *actb1* and *rps18* for normalization of relative gene expression of candidate target genes.

**Table 1:** Ranking and statistical analyses of reference genes in the caudal fin of *L. tigripictilis* using three different algorithms

BestKeeper				geNorm		NormFinder	
Ranking	SD	Ranking	r	Ranking	M	Ranking	SV
*rps18*	0.975	*rps18*	0.732	*actb1*	0.530	*actb1*	0.183
*actb1*	0.973	*actb1*	0.903	*rps18*	0.548	*rps18*	0.229
*hsp90a*	0.958	*hsp90a*	0.946	*hsp90a*	0.562	*rps11*	0.246
*rps11*	0.942	*rps11*	0.992	*rps11*	0.604	*hsp90a*	0.254
*tbp*	0.902	*hprt1*	1.013	*hprt1*	0.685	*tbp*	0.377
*hprt1*	0.889	*tbp*	1.061	*tbp*	0.690	*hprt1*	0.418
*elf1a*	0.798	*elf1a*	1.197	*elf1a*	0.904	*elf1a*	0.512
*gapdh*	0.752	*gapdh*	1.212	*gapdh*	0.976	*gapdh*	0.540

*SD* standard deviation, *r* Pearson correlation coefficient, *SV* stability value, *M* M value of stability

### Primer design

In order to design qPCR primers, we aligned the orthologues of each gene from different African cichlid tribes including one species of Tilapiini (*Oreochromis niloticus;* Linnaeus, 1758), one species of Lamprologini (*N. brichardi*), and one species of Haplochromini (*Astatotilapia burtoni*; Günther, 1894) (Brawand et al., 2014; Santos et al., 2016; Singh et al., 2017). The 1-to-1 orthologues were confirmed by blasting zebrafish mRNA REfSeq IDs against *N. brichardi* transcriptome in NCBI and cross-checking the top hits returned by BLAST in the Ensembl database for zebrafish and *O. niloticus* orthologues (http://www.ensembl.org). Next, we used the aligned sequences to identify conserved regions across the species (using CLC Genomic Workbench, CLC Bio, Denmark) and at the exon/exon boundaries (using annotated genome of *O. niloticus* in the Ensembl database. Primers with short amplicon sizes (< 250 bp) were designed using Primer Express 3.0 (Applied Biosystems, CA, USA) and OligoAnalyzer 3.1 (Integrated DNA Technology) (Supplementary data 1), as previously described (Ahi et al., 2020).

### RNA isolation and real-time qPCR

RNA was isolated from individual tissue samples using the Trizol protocol described in (Ahi et al., 2017). DNA was removed enzymatically and RNA concentration was measured by spectrophotometry using a nanophotometer (IMPLEN GmbH, Munich, Germany). RNA quality (integrity number > 7) was ascertained in a R6K ScreenTape System on an Agilent 2200 TapeStation (Agilent Technologies). cDNA was prepared from 1000 ng of RNA using the High Capacity cDNA Reverse Transcription kit (Applied Biosystems), according to the manufacturer's protocol. Negative controls, i.e., reactions without addition of reverse transcriptase (-RT samples), were prepared to confirm the absence of genomic DNA. cDNA was diluted 1:3 times in nuclease-free water for further use in quantitative real-time PCR.

The qPCR was conducted using Maxima SYBR Green/ROX qPCR Master Mix (2×) by following the manufacturer’s instruction (Thermo Fisher Scientific, St Leon-Rot, Germany) in 96-well PCR plates on an ABI 7500 real-time PCR System (Applied Biosystems). The experimental set-up per run followed the preferred sample maximization method (Hellemans et al., 2007). The primer efficiency analyses in LinRegPCR v11.0 (http://LinRegPCR.nl) (Ramakers et al., 2003) were conducted as described in our previous study (Ahi et al., 2017).

### Analysis of qPCR data

The geometric mean of the Cq values (Vandesompele et al., 2002) of the two reference genes Cq_reference_ was used to normalize Cq values of target genes in each sample (ΔCq_target_ = Cq_target_ – Cq_reference_). We randomly selected one biological replicate of the dorsal region of the caudal fin (stage 0) as calibrator sample, and in order to calculate ΔΔCq values, we subtracted the ΔCq values of all samples from the calibrator ΔCq value (ΔCq_target_ – ΔCq_calibrator_). Relative expression quantities (RQ values) were calculated as 2^−ΔΔCq^ (Pfaffl, 2001).

**Fig. 2:**
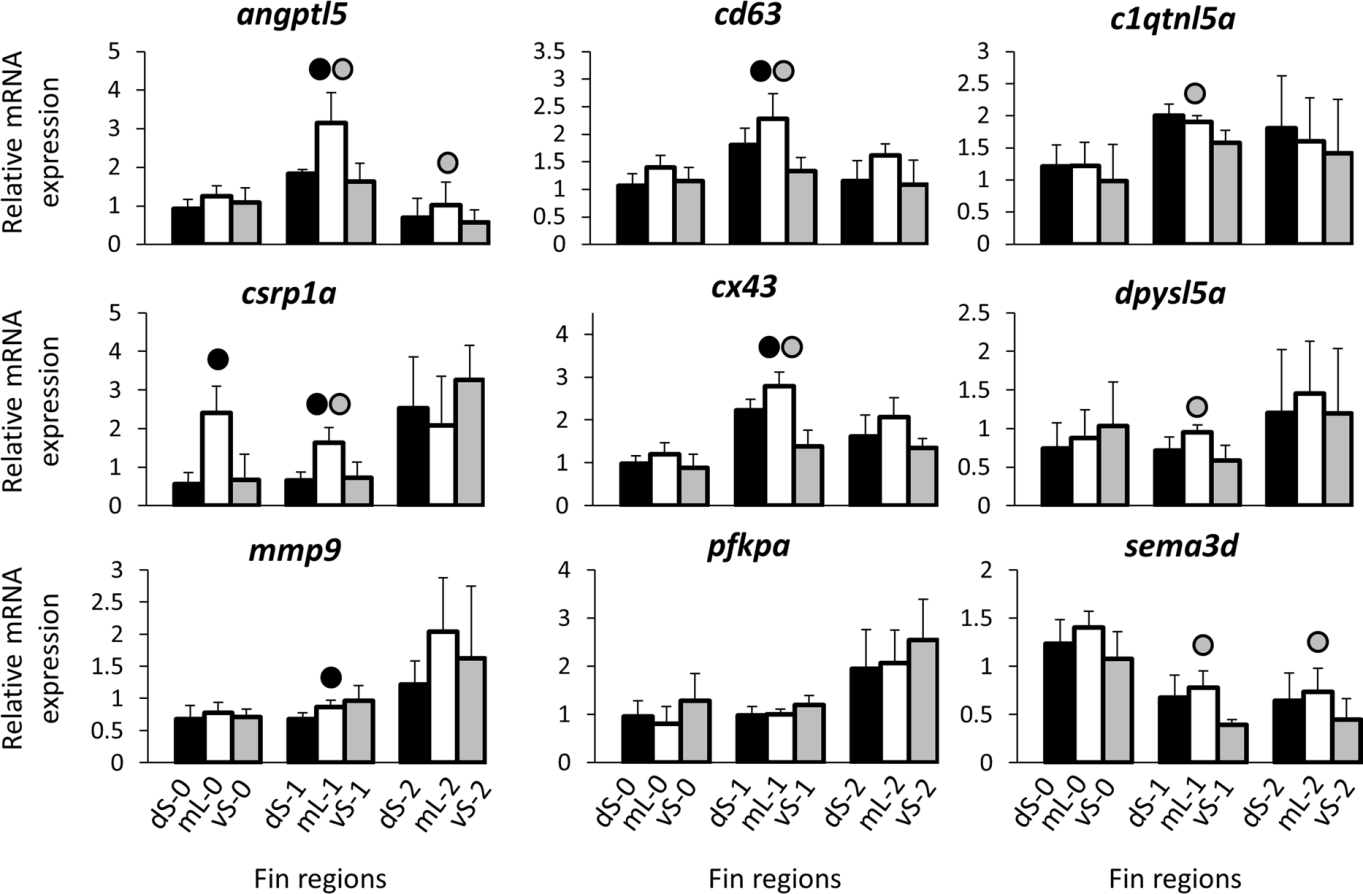
Expression levels of candidate genes selected based on already identified GRN in *N. brichardi*. Means and standard deviations of RQ in three biological replicates are shown for the elongated and short regions of the caudal in original (stage 0) and regenerating tissue. See Fig. 1A for fin region codes; numbers 0 to 2 identify regeneration stages. Circles above bars indicate significantly elevated expression (*P* < 0.05 in paired t-tests; Supplementary data 2, Table S3) in comparisons between elongated and short fin region samples (i.e., compared to the bar matching the shade of the circle)

**Fig. 3:**
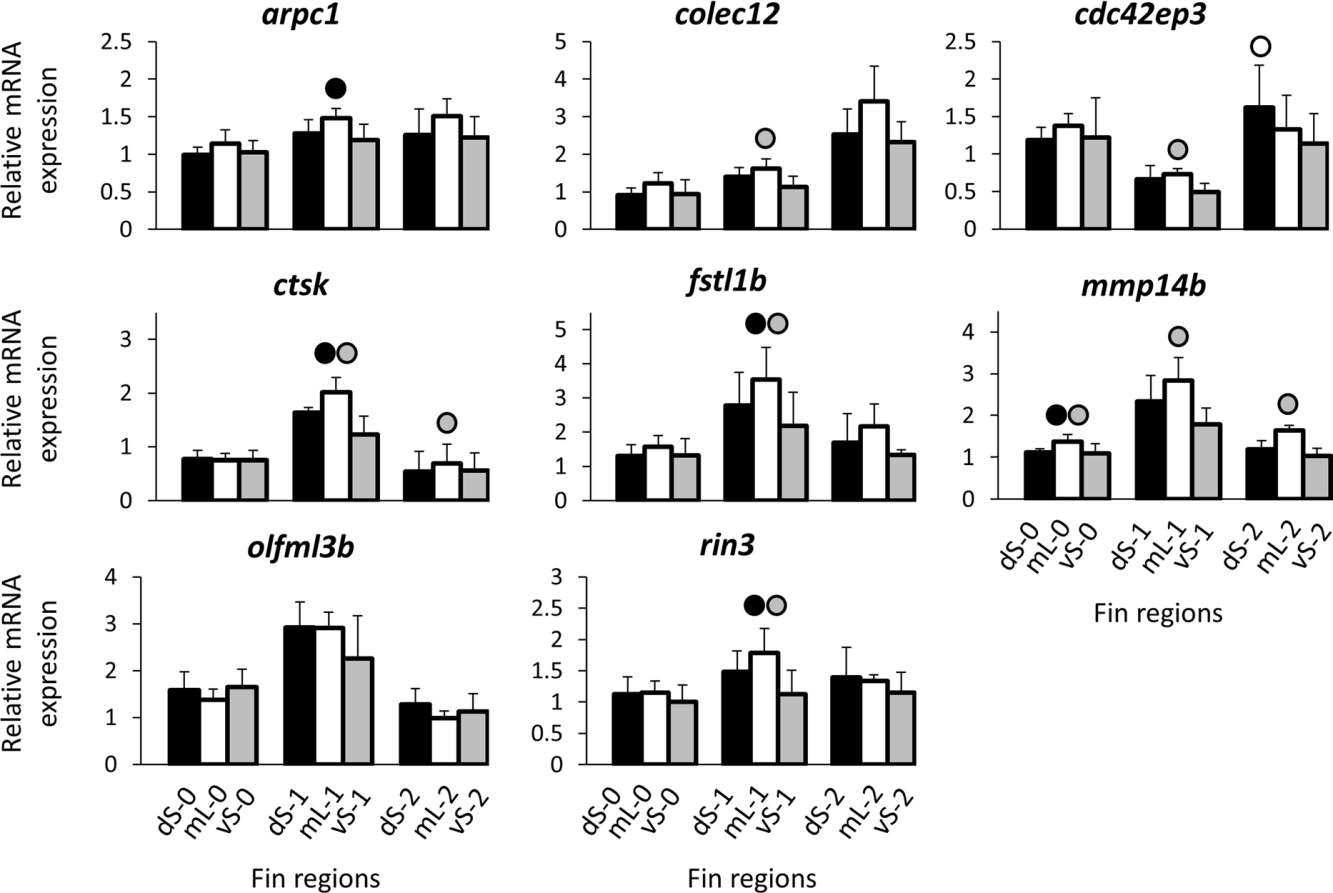
Expression levels of candidate genes selected based on co-expression with *csrp1a*, *angptl5*, and *cd63*. Means and standard deviations of RQ in three biological replicates are shown for the elongated and short regions of the caudal in original (stage 0) and regenerating tissue. See Fig. 1A for fin region codes; numbers 0 to 2 identify regeneration stages. Circles above bars indicate significantly elevated expression (*P* < 0.05 in paired *t*-tests; Supplementary data 2, Table S3) in comparisons between elongated and short fin region samples (i.e., compared to the bar matching the shade of the circle)

**Fig. 4:**
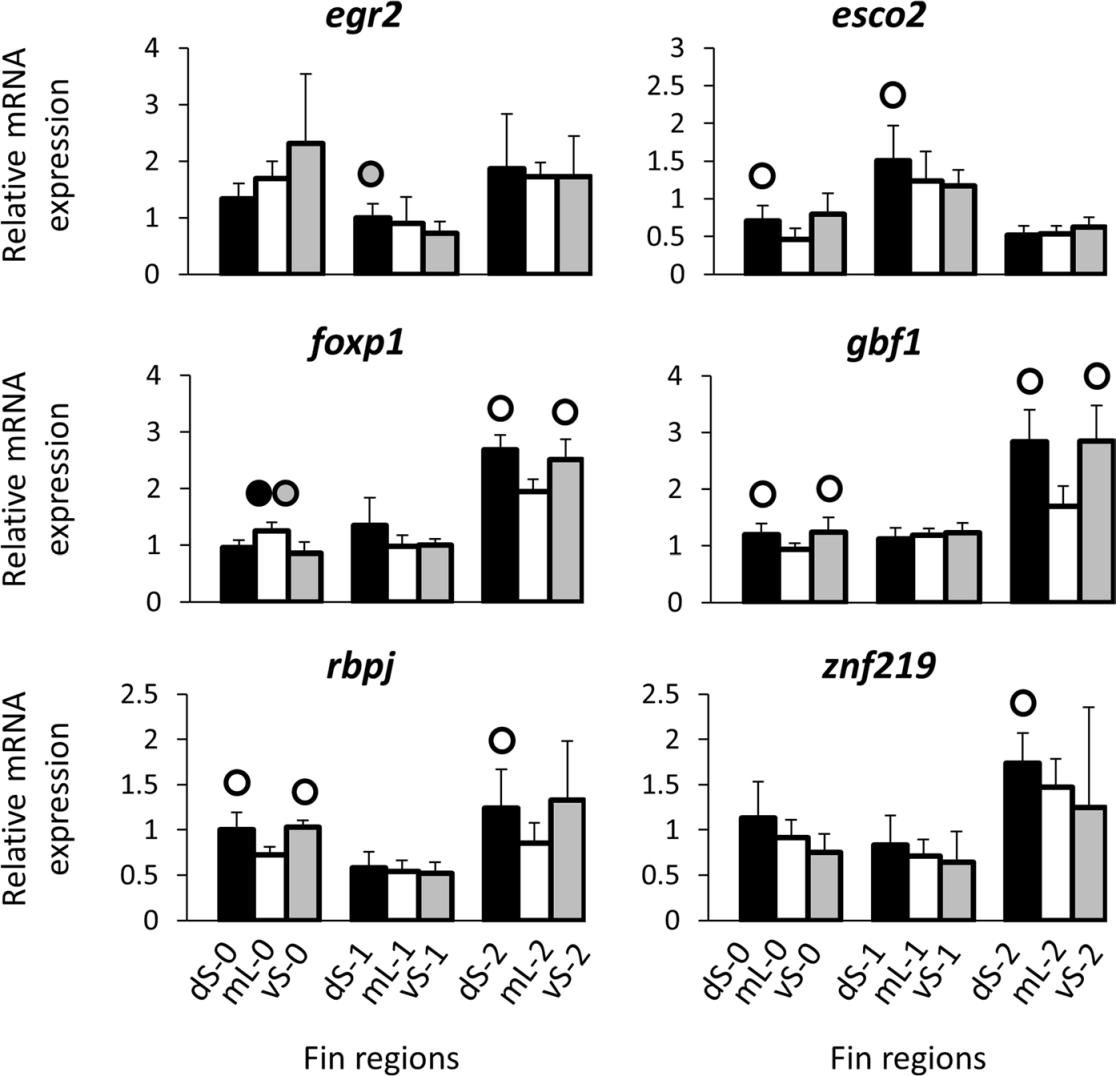
Expression levels of predicted upstream regulators. Means and standard deviations of RQ in three biological replicates are shown for the elongated and short regions of the caudal in original (stage 0) and regenerating tissue. See Fig. 1A for fin region codes; numbers 0 to 2 identify regeneration stages. Circles above bars indicate significantly elevated expression (*P* < 0.05 in paired *t*-tests; Supplementary data 2, Table S3) in comparisons between elongated and short fin region samples (i.e., compared to the bar matching the shade of the circle)

RQ values were log-transformed for statistical analyses. For each target gene, differences in expression levels between dorsal (short) and medial (elongated), ventral (short) and medial (elongated), as well as dorsal (short) and ventral (short) regions of the caudal fin were tested in linear mixed models with log(RQ) as dependent variable, fin region as fixed factor and developmental stage nested within biological replicate (fish) as random factors (Supplementary data 2, Table S2). To account for multiple testing (*N* = 111 comparisons; 37 candidate genes times 3 fin region contrasts), *P* values for the effect of length were corrected using the Benjamini–Hochberg procedure (Benjamini & Hochberg, 1995). We fit analogous linear mixed models including only stage 1 and stage 2 data (i.e., the regenerating tissue but not the intact tissue). The log-transformed RQ values were also used for calculations of pairwise Pearson correlation coefficients (*r*) among the candidate genes. Finally, paired *t*-tests were used to conduct stage-specific comparisons between fin regions, again using log-transformed RQ values. Bonferroni–Hochberg corrections of p-values were conducted for multiple testing within each of the fin region contrasts at each stage (*N* = number of genes = 37), and corrected p-values were used to mark significant differences in the barplot figures illustrating gene expression levels (Figs. 2, 3, 4; see also Supplementary Data 2, Table S3).

## Results

### Anatomical characterization of caudal fin rays of *L. tigripictilis* and *N. brichardi*

The lengths of the fin ray segments (Fig. 1; Supplementary data 2, Table S1) did not differ significantly between the elongated, medial fin region (mean ± sd = 309.4 ± 25.5 µm) and the short, ventral fin region (mean ± sd = 315.1 ± 25.2 µm; est. = 5.9, *t* = 1.6, *P* = 0.12). In contrast, the individual fin ray segments were significantly longer in the short, dorsal fin region (mean ± sd = 339.6 ± 28.6 µm), both compared to the medial fin region (est. = 30.5, *t* = 8.2, *P* = 5.6 × 10^–10^) and compared to the ventral fin region (est. = 24.6, *t* = 5.7, *P* = 1.2 × 10^–6^; complete model output is reported in Supplementary data 2, Table S1). Hence, comparisons between the medial and the ventral region of the caudal fin of *L. tigripictilis* represent a contrast between long and short fin rays that differ in the number of segments per ray. In contrast, comparisons involving the dorsal fin region involve differences in both segment length (longer in dS) and number (fewer in dS), with a larger difference in the number of segments between dS and mL than between dS and vS.

To enable a comparison with *N. brichardi* (i.e., the cichlid species, in which the GRN was originally reconstructed), we re-analyzed the fin ray segment length data of the caudal fin of *N. brichardi* (data from Ahi et al., 2017). In the original analysis (Ahi et al., 2017), data from the dorsal and the ventral edges of the caudal fin had been pooled for comparison with the medial region and no difference in segment length was detected. As for *L. tigripictilis*, we now distinguished between the dorsal elongated and the ventral elongated rays of the caudal fin of *N. brichardi* and compared each to the medial short rays. This analysis revealed that the segments of the (long) dorsal rays of the caudal fin of *N. brichardi* were longer than those of the (long) ventral rays (mean ± SL = 289.9 ± 54.3 µm, in the dorsal region; 238.7 ± 33.0 µm, in the ventral region; *N* = 5 segments of 2 rays from each of 3 fish per fin region; LM, est. = 53.5, *t* = 2.3, *P* = 0.05), but in contrast to *L. tigripictilis*, the segments in the (short) medial rays were equally long as those in the (long) dorsal rays (mean ± SL = 291.8 ± 29.0 µm, in the medial region, *N* = 5 segments of 2 rays from each of 4 fish; LM, est. = 2.1, *t* = 0.2, *P* = 0.87).

### Expression analysis of candidate genes

Each of the three pairwise comparisons between fin regions represents a different phenotypic contrast (see above), and we therefore conducted three pairwise comparisons of candidate gene expression levels among the fin regions (as opposed to combining the data into a single ‘short’ versus ‘long’ comparison). In the following text, ‘expression in the medial elongated region of the caudal fin’ is abbreviated as ‘mL expression,’ ‘expression in the dorsal region’ is abbreviated as ‘dS expression,’ ‘expression in the ventral region’ is abbreviated as ‘vS expression,’ and the expression levels are reported as ‘increased’ or ‘decreased’ in comparison with the other regions. We obtained similar results in analyses that included all three stages (intact fin and regeneration stages 1 and 2) and in analyses restricted to the two regeneration stages and reported the analyses of the full data unless noted otherwise. Table 2 summarizes the significant expression level differences detected among the tested candidate genes, and the complete statistical analyses are reported in the supplementary material (Supplementary data 2, Table S2).

**Table 2:** Summary of anatomical and gene expression patterns in the caudal fin regions of *L. tigripictilis*, compared to the patterns reported in two other cichlid species

Fin ray segment lengths differences			
*L. tigripictilis*	*N. brichardi*	*S. casuarius* (dorsal fin)	*S. casuarius* (anal fin)
dS > mL = vS	Anal and dorsal fin: S > L Caudal fin: L_dorsal_ = S_medial_ > L_ventral_	L > S	L > S

Gene expression differences					
Genes	Selection	Expression	*N. brichardi*	*S. casuarius* (dorsal fin)	*S. casuarius* (anal fin)
Elevated mL expression in *L. tigripictilis*					
angptl5*^#^	*N.b.*-GRN	mL > dS = vS	L	L	L
cd63*^#^	*N.b.*-GRN	mL > dS = vS	L	L	No diff
csrp1a*^#^	*N.b.*-GRN	mL > dS = vS; stage 0	L	No diff	No diff
*cx43*^*#*^	*N.b.*-GRN	mL > dS > vS	L	L	L
*sema3d*	*N.b.*-GRN	mL > dS > vS	S	S	S
*colec12*^*#*^	co-exp	mL > dS > vS	–	–	–
*mmp14b*^*#*^	co-exp	mL > dS > vS	–	–	–
*arpc1*^*#*^	co-exp	mL > dS = vS	–	–	–
*f* *stl1b*^*#*^	co-exp	mL > dS = vS	–	–	–
Reduced mL expression in *L. tigripictilis*					
*gbf1*	TF	dS = vS > mL	–	–	–
*rbpj*	TF	dS = vS > mL	–	–	–
*esco2*	TF	dS = vS > mL; stage 0	L (caudal and dorsal fins)	No diff	No diff
Reduced vS expression in *L. tigripictilis*					
*c1qtnl5*	*N.b.*-GRN	mL = dS > vS	L	No diff	No diff
*cdc42ep3*	co-exp	mL = dS > vS	–	–	–
*rin3*	co-exp	mL = dS > vS	–	–	–
Elevated vS expression in *L. tigripictilis*					
*pfkpa*	*N.b.*-GRN	vS > mL = dS; stage 0	L	S	No diff
Reduced dS expression in *L. tigripictilis*					
*mmp9*	*N.b.*-GRN	mL = vS > dS	L	S	No diff
Differences involving only two of the fin regions in *L. tigripictilis*					
*dpysl5a*	*N.b.*-GRN	mL > vS; stage 1 and 2	L	No diff	No diff
*ctsk*	co-exp	mL > vS	–	–	–
*alx4a*	TF	dS > vS; stage 0	–	–	–
*znf219*	TF	dS > vS; stage 0	–	–	–
*f* *oxp1*	TF	dS > mL; stage 1 and 2	S	No diff	No diff

Data on *N. brichardi* are from Ahi et al. (2017) and Ahi & Sefc (2018); data on *S. casuarius* are from Ahi et al. (2019a). mL, dS and vS are the medial long, dorsal short, and ventral short regions of the caudal fin of *L. tigripictilis*, while L and S are the elongated and short regions of the fin types examined in *N. brichardi* and *S. casuarius*. In the summary of the segment length differences, dS > mL (for instance) indicates that segments in the dS region are longer than those in the mL region. In the summary of the gene expression differences, we report the results for genes with significant expression level differences detected in *L. tigripictilis*, sorted by the detected pattern. ‘*N.b.*-GRN’ identifies candidate genes that are part of the gene regulatory network identified in *N. brichardi*; ‘co-exp.’ identifies candidate genes based on co-expression; ‘TF’ identifies the predicted transcription factors. Asterisks mark genes underlying the search for co-expressed candidate genes; hashes mark genes used for TF prediction. The gene expression pattern mL > dS = vS for *angptl5*, for instance, signifies that the expression level of the gene is significantly higher in mL compared to dS and to vS, whereas expression levels in dS and vS are not significantly different from each other. Unless developmental stages are indicated, the reported difference in gene expression levels was observed in the analysis including all stages. “S” and “L” stand for significantly elevated gene expression levels in short or long, respectively, regions of the fins of *N. brichardi* and *S. casuarius* detected in previous studies. In *N. brichardi*, the expression patterns were largely consistent across the three fin types and also within the caudal fin (i.e., concerning the contrasts between the medial short region on the one hand and the dorsal and ventral elongated regions on the other hand); therefore, results were summarized across fins unless indicated otherwise. ‘no diff.’ indicates that no significant expression L/S differences could be detected in *N. brichardi* or *S. casuarius*; when no pattern is reported for *N. brichardi* and *S. casuarius*, these genes were not tested in these species

In the first step of our gene expression analysis, we examined the expression levels of 16 members of the *N.b.*-GRN. Among these, we detected increased mL expression compared to both vS- and dS expression for *angptl5*, *cd63*, *csrp1a*, *cx43*, and *sema3d*, and increased mL expression compared to vS expression for *c1qtnf5* and *dpysl5a* (mainly in stage 1 of fin regeneration) (Table 2, Fig. 2; Supplementary data 2, Table S2). Additionally, dS expression of *mmp9* was lower than mL expression. We also detected significant expression level differences for *pfkpa* (higher mL- than dS expression during regeneration).

The second step of our analysis was based on the three genes, *angptl5*, *cd63*, and *csrp1a*, which had the strongest expression differences between the elongated medial and the short fin regions in the above analysis. Using the zebrafish co-expression database, we identified eight additional candidate genes that are co-expressed with each of these genes and compared their expression levels between the caudal fin regions of *L. tigripictilis* (*arpc1*, *cdc42ep3*, *colec12*, *ctsk*, *fstl1b*, *mmp14b*, *olfml3b*, and *rin3;* Supplementary data 1). Among these, increased mL expression compared to both vS and dS expressions was detected for *colec12*, *ctsk*, *fstl1b*, *mmp14b*, and *rin3*, whereas increased mL expression compared to only one of the short regions (vS or dS) was detected for *arpc1 and cdc42ep3*, and no expression difference was detected for *olfml3b* (Table 2, Fig. 3; Supplementary Data 2, Table S2).

### Expression analysis of candidate upstream regulators

Predicted upstream regulators for eight genes with the most strongly increased mL expression in the above analyses (*angptl5*, *cd63*, *ctsk*, *cx43*, *csrp1a*, *fstl1b*, *mmp14b*, and *rin3*) included the 12 transcription factors *alx4a*, *ap4*, *egr1*, *egr2*, *foxd3*, *foxp1*, *gbf1*, *heb*, *patz1*, *rbpj*, *srf*, and *znf219* (Supplementary Data 1). Additionally, we tested the expression of *esco2* (which was not among the predicted TFs) because it regulates *cx43* and *sema3d* in a gene regulatory network involved in the formation, growth, and regeneration of fin ray segments and joints (Iovine et al., 2005; Govindan & Iovine, 2014, 2015; Banerji et al., 2016; Govindan et al., 2016). Among the 13 tested TFs, decreased mL expression (compared to both vS and dS expressions) was detected for *gbf1*, *esco2*, and *rbpj* when data of all three developmental stages were pooled, but not when only stage 1 and 2 (regeneration) were analyzed. Finally, *znf219* and *alx4a* showed higher dS than vS expression, but only in analyses across all three developmental stages (Table 2, Fig. 4; Supplementary data 2, Table S2).

### Gene expression correlations

**Fig. 5:**
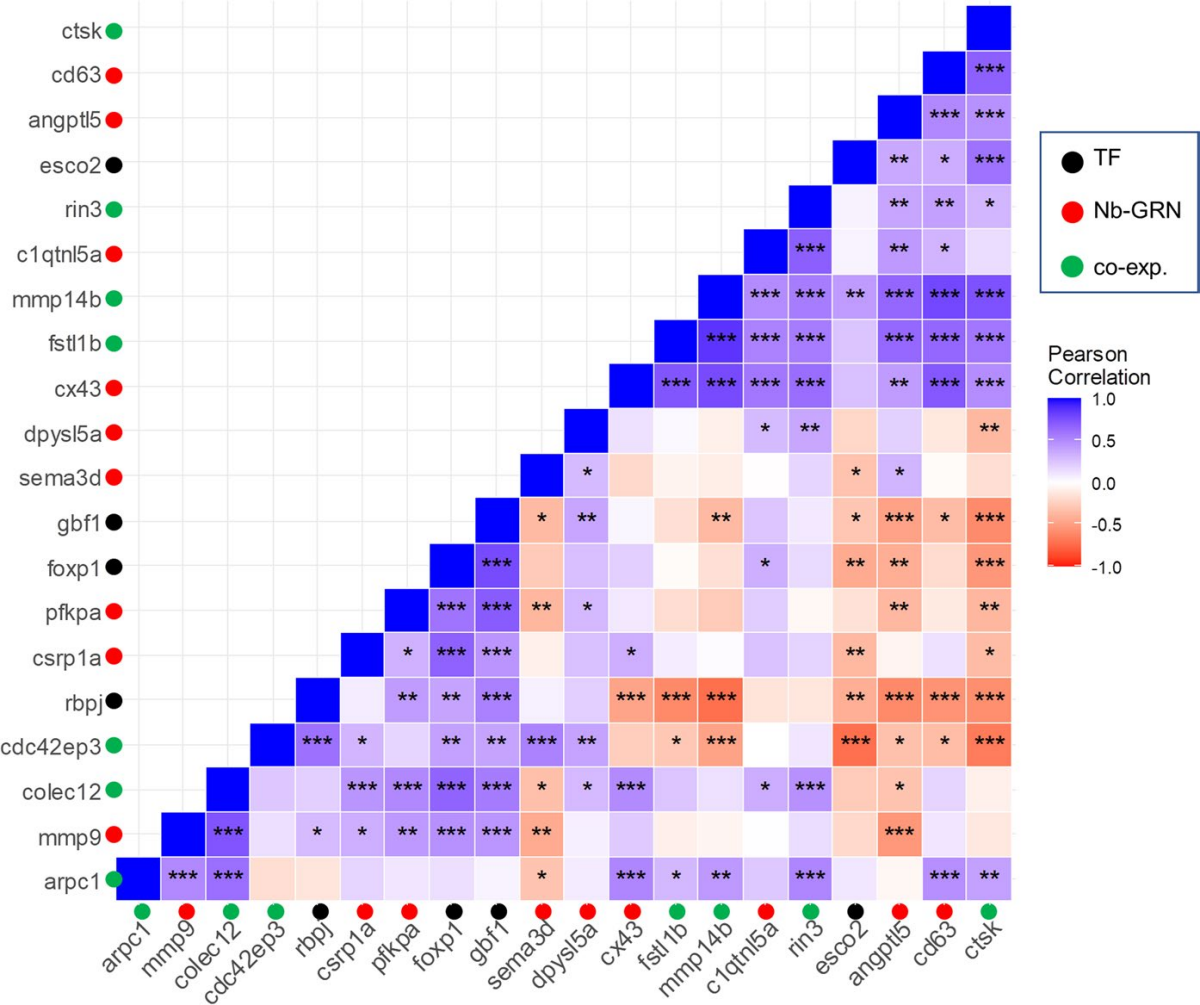
Correlation analysis reveals significant positive or negative co-expression of the candidate genes. Pearson correlation coefficient (*r*) was used to assess the pairwise expression similarity between the candidate genes during craniofacial development. Blue represents positive and red represents negative expression correlation. *P < 0.05; **P < 0.01; ***P < 0.001

We tested for expression correlations among those of the candidate genes and upstream regulators, which had shown significant expression differences between elongated and short fin regions. A number of significant pairwise correlations as well as clusters of correlated genes were detected (Fig. 5). For instance, a cluster of positively correlated gene expression levels comprised the genes *cx43*, *fstl1b*, *mmp14b*, *c1qtnl5a*, *rin3*, *angptl5*, *cd63*, and *ctsk*. These genes showed higher expression in the mL region in comparison with one or both of the short fin regions (dS and/or vS). The expression levels of these genes were negatively correlated with that of the transcription factor *rbpj* and positively with that of the TF *esco2*. We also detected strong positive expression correlations among the transcription factors *foxp1*, *gbf1*, and *rbpj*. Expression of each of these TFs was negatively correlated with the expression of the TF *esco2*, and positively correlated with the expression of *pfkpa*, *colec12*, *cdc42ep3*, and *dpysl5a*.

## Discussion

Convergence across species in a morphological trait may, but need not, coincide with shared mechanisms at an anatomical and genetic level (Chan et al., 2010; Elmer & Meyer, 2011; Colombo et al., 2013; Conith et al., 2019). The present study addressed the genetic control of the differential growth of fin regions, which underlies the formation of the fin shape, in a cichlid fish species. If mechanisms of fin shape formation were strongly conserved, we would expect congruent gene expression patterns in elongated compared to short fin regions, both across types of fins and across species. The expression patterns of some of the *N.b.*-GRN genes that had previously been found to be associated with fin shape in the cichlid fish *N. brichardi* were indeed correlated with fin shape in the present focal species, *L. tigripictilis*. Many of them, however, were not, and some of the expression patterns and inferred regulatory interactions differed from *N. brichardi*, which suggests a certain level of divergence in the pathways controlling fin shape between related cichlid species. We therefore searched for further candidate genes and upstream regulators that might be involved in fin shape formation in *L. tigripictilis* and detected several promising candidates. In the following, we first discuss fin shape-associated expression patterns of the *N.b.*-GRN genes in the focal species, *L. tigripictilis*, in relation to available data from two other cichlid species. We then attend to the new candidate genes brought forth in this study and review published data that support their role in fin growth.

### Expression patterns of the *N.b.*-GRN genes in *L. tigripictilis* fins

Slightly more than half of the *N.b.*-GRN members showed significant expression level differences between the fin regions of *L. trigripictilis*, but not all of these expression differences were in the direction predicted based on the patterns observed in *N. brichardi* (Ahi et al., 2017, Ahi & Sefc, 2018; summarized in Table 2). Fin shape-associated expression levels of the *N.b.*-GRN member genes have previously also been examined in the cichlid *S. casuarius* (Ahi et al., 2019a), and the set of genes with significant expression differences shared between *N. brichardi* and *L. tigripictilis* in the present study overlapped only partially with the genes that had shared expression differences between *N. brichardi* and *S. casuarius* (Ahi et al., 2019a; summarized in Table 2). An interesting congruence across all three species was detected for *mmp9*, which encodes a matrix remodeling enzyme with a role in fin regeneration (Yoshinari et al., 2009; LeBert et al., 2015). Expression of *mmp9* was consistently lowest in those fin regions with the longest ray segments (i.e., the dorsal short region in *L. tigripictilis*, the short fin regions in *N. brichardi* and the elongated fin regions in *S. casuarius*; Table 2), suggesting that *mmp9* expression might be associated with reduced ray segment length in the three cichlid species. In zebrafish, *mmp9* is negatively regulated by *cx43* (Ton & Iovine, 2013), which encodes a subunit of the gap junction protein complex and whose expression is positively correlated with segment length in the fin rays of zebrafish (Iovine et al., 2005; Sims et al., 2009). *cx43* is a member of the *N.b.*-GRN and showed elevated expression in the fin regions with shorter segments in the *L. tigripictilis* and *N. brichardi*, suggesting that the interaction between *cx43* and *mmp9* and the role of *cx43* in segment growth may differ between the zebrafish and the lamprologine cichlids. Regardless of segment length, however, *cx43* was consistently more strongly expressed in the elongated fin regions of each of the three cichlid species (Table 2), which corroborates the gene as a strong candidate for a regulator of fin growth.

In zebrafish, *cx43* is also a positive regulator of another of the tested *N.b.*-GRN genes, *sema3d*, which mediates the *cx43*-dependent functions in cell proliferation, joint formation, and phenotypic changes of zebrafish fins (Ton & Iovine, 2012). In humans, mutations in *cx43* and *sema3d* are associated with defects in finger growth [brachydactyly, Kjaer et al., 2004; Jamsheer et al., 2014); ectrodactyly (Sivasankaran et al., 2015)]. The elevated mL expression of both *sema3d* and *cx43* in *L. tigripictilis* is therefore concordant with known roles of these genes in skeletal growth. However, the functional relationship between the two genes appears to be different in *N. brichardi* and *S. casuarius*, where *sema3d* and *cx43* showed opposite expression differences between L and S fin regions (Table 2).

Increased expression in elongated fin regions across all three studied cichlid species was detected for *cd63*, which induces spinal cord regeneration in the axolotl (Monaghan et al., 2006), and for *angptl5*, which encodes an angiopoietic protein. Increased expression of *angptl5* was also observed during exaggerated elongation of the caudal fin in swordtail fish (Kang et al., 2015). In humans, the expression of *angptl5* in endothelial cells is highly induced through interaction with osteoblasts during osteogenesis and bone remodeling (Simunovic et al., 2013). These data indicate that *angptl5* expression during skeletal outgrowth is a marker for induced angiogenesis in the skeletal tissue across vertebrate taxa.

In summary, the present data collated with previous results (Ahi & Sefc, 2018; Ahi et al., 2019a) highlight a set of genes, including *angptl5*, *cd63*, *cx43,* and *mmp9*, whose expression levels were consistently correlated with fin elongation across three cichlid species. However, numerous differences between species with respect to which gene expression levels co-varied with fin shape and how gene expression levels were correlated with one another suggest that networks of genes involved in fin shape formation are not fully congruent between species. Our data also indicate variation in regulatory interactions, as only one of the upstream regulators of the *N.b.*-GRN, *foxp1*, displayed significant expression differences among fin regions in *L. tigripictilis.* Since the investigated fin types and their shapes differed between the three cichlid species, some of the inter-specific incongruences in gene expression patterns may reflect positional effects, for instance, when different genes control the growth of medial fin regions compared to elongations of the dorsal and ventral edges. Some of the incongruent gene expression patterns are likely associated with differences in the anatomical basis of fin ray elongation, specifically the lengths of fin ray segments in short and elongated fin regions. In the two lamprologine cichlid species, segment length variation was decoupled from fin ray length, whereas in *S. casuarius*, longer rays consisted of longer segments than shorter rays (Table 2,2). Consequently, expression patterns of some of the tested genes are expected to differ between species contingent on whether the gene controls the length (as, for instance, indicated for *mmp9*) or the number of ray segments, and functional studies are needed to elucidate their possible functions in fin growth.

### Novel fin shape candidate genes detected in *L. tigripictilis*

Given that our data linked only few of the original candidate genes from the *N.b.*-GRN to fin shape formation in *L. tigripicilis*, we extended our candidate gene set based on existing co-expression data in zebrafish. Seven of the new candidate genes showed gene expression differences between the fin regions of *L. tigripictilis* (Table 2). Three of the new genes, *fstl1, colec12*, and *mmp14*, have already been implicated in skeletal morphogenesis in mammals or zebrafish. Follistatin-like 1, *fstl1*, encodes a potent antagonist of the BMP pathway during skeletogenesis in vertebrates (Sylva et al., 2011) and has been shown to affect digit formation in mammals (Lorda-Diez et al., 2013; Sylva et al., 2013). *Colec12* (previously known as collectin placenta protein 1 gene, *clp1*) encodes collectin-12, which is involved in vasculogenesis and has been shown to be positively associated with body elongation in zebrafish during development; i.e., knockdown of *colec12* caused shortened body length in zebrafish (Fukuda et al., 2011). The metalloprotease encoding *mmp14* gene is involved in human skeletogenesis with effects on finger and toe morphology (Wilkinson et al., 2012; De Vos et al., 2018, 2019). In zebrafish, mutations in *mmp14b* can lead to skeletal anomalies including shortening of body and fin, prominent frontal bone and skeletal curvatures (De Vos et al., 2018). The elevated expression levels of *mmp14* and *colec12* in elongated compared to short fin regions in *L. tigripictilis* are consistent with the mutant zebrafish phenotypes.

We also extended our candidate gene set by predicting transcription factors for genes with strongly increased mL expression in *L. tigripictilis*. TFs predicted from genes with strongly increased mL expression are expected to display expression correlations with these genes and therefore to display either increased or decreased mL expression. The expected pattern was observed for *gbf1* and *rbpj*, both of which showed reduced mL expression compared to both dS and vS fin regions. The expression levels of *gbf1* and *rbpj* were also significantly correlated with the majority of the genes involved in the prediction procedure (Fig. 5). Golgi brefeldin A-resistant factor 1 gene, *gbf1*, encodes a protein that functions as a guanine nucleotide exchange factor and plays important roles in regulating organelle structure and cargo-selective vesicle trafficking (Manolea et al., 2008). During zebrafish development, *gbf1* is involved in vascular system formation, pigmentation, and morphogenesis of the caudal fin (Chen et al., 2017). Knockdown of *GBF1* in mammalian cells leads to a range of structural anomalies which eventually inhibit trafficking of transmembrane proteins and cell death (Citterio et al., 2008).

The second TF, *rbpj*, encodes a transcription factor with dual regulatory activities, which can regulate skeletogenic process both as an activator and as a repressor, depending on its interaction with Notch signal proteins (Castel et al., 2013). For instance, the Notch intracellular domain (NICD) and *rbpj* form a complex that can act as transcription repressor and negatively regulate chondrocyte differentiation (Chen et al., 2013). On the other hand, the NICD-rbpj complex acts as transcriptional activator inducing osteoblast proliferation (Tao et al., 2010). Moreover, *rbpj* has been shown to inhibit osteoclastogenesis and bone resorption (Zhao et al., 2012; Miller et al., 2016). It should be noted that Notch signal activity and *rbpj* transcription are both required for maintaining blastema cells in a plastic, undifferentiated, and proliferative state, which is essential for fin regeneration in zebrafish (Münch et al., 2013). In human, mutation in *Rbpj* is shown to be associated with etiology of Adams-Oliver syndrome (AOS) which is identified with multiple-malformation disorders, and particularly, with terminal limb defects (Hassed et al., 2012; Nakayama et al., 2014). The terminal limb defects in AOS are characterized by shortening of the end of the fingers (brachydactyly) and curvature of the digits (clinodactyly) (Nakayama et al., 2014). In mammals, *rbpj* can act as upstream regulator of *mmp14* expression (Gao et al., 2016; Nus et al., 2016). The two genes show opposite expression patterns (*mmp14* with elevated and *rbpj* with reduced mL expression, respectively) and significantly negative expression correlations (*r* = − 0.72, *P* > 0.001) in *L. tigripictilis*, suggesting that a regulatory interaction also exists in this cichlid species.

Finally, expression correlations suggested that the TF *esco2* is connected with several of the tested TFs and genes, notably *angptl5* and *cd63*, which our data strongly implicated in fin elongation. In contrast to evidence from zebrafish (Banerji et al., 2016), where *esco2* is a positive regulator of *cx43*, there was no evidence for a regulatory link between *esco2* and *cx43* in *L. tigripictilis*. This is consistent with previous data from *N. brichardi* (Ahi and Sefc 2018), but opposite to *S. casuarius*, where expression levels of *esco2* were positively correlated with those of *cx43*. These findings suggest a role for *esco2* in the formation of fin shape in cichlid fish, but divergence with respect to the underlying molecular mechanisms.

## Conclusions

The present study reports genes and transcription factors, whose expression levels were statistically linked to fin shape in the cichlid species *L. tigripitilis*. Among the tested candidate genes were members of a GRN that had previously been proposed to be involved in the filamentous elongation of fin edges in another cichlid species, *N. brichardi*. Only few of the *N.b.*-GRN genes showed the expected expression patterns in elongated and short regions of the *L. tigripictilis* caudal fin. Moreover, the predicted upstream regulators of the genes, whose expression was statistically associated with elongated fin regions, were also different in *L. tigripictilis* (*gbf1* and *rbpj*) from those found in *N. brichardi*. Considering gene functions and interactions known in zebrafish as well as the gene expression data from previous work on cichlid fishes, the present data demonstrate some degree of conservation, but also substantial differences in the regulation of fin growth among cichlid fishes and also in comparison with zebrafish.

## Supplementary Information

Below is the link to the electronic supplementary material.

Supplementary data 1. Primer information, selection of co-expressed genes and TF binding site enrichments (XLS 113 kb)

Supplementary data 2 Statistical analyses of fin ray segment length and gene expression data (XLSX 38 kb)

Supplementary data 3. Data generated in this study (XLSX 36 kb)

## Data Availability

All data generated during this study are included in the supplementary material accompanying this published article.
